# Rope-based oral fluid sampling for early detection of classical swine fever in domestic pigs at group level

**DOI:** 10.1186/s12917-016-0930-2

**Published:** 2017-01-05

**Authors:** Klaas Dietze, Anna Tucakov, Tatjana Engel, Sabine Wirtz, Klaus Depner, Anja Globig, Robert Kammerer, Susan Mouchantat

**Affiliations:** Friedrich-Loeffler-Institut, Federal Research Institute for Animal Health, Südufer 10, 17493 Greifswald Insel Riems, Germany

**Keywords:** Non-invasive, Rope-in-a-bait, Surveillance, Hog cholera, Group level, Backyard pig production

## Abstract

**Background:**

Non-invasive sampling techniques based on the analysis of oral fluid specimen have gained substantial importance in the field of swine herd management. Methodological advances have a focus on endemic viral diseases in commercial pig production. More recently, these approaches have been adapted to non-invasive sampling of wild boar for transboundary animal disease detection for which these effective population level sampling methods have not been available. In this study, a rope-in-a-bait based oral fluid sampling technique was tested to detect classical swine fever virus nucleic acid shedding from experimentally infected domestic pigs.

**Results:**

Separated in two groups treated identically, the course of the infection was slightly differing in terms of onset of the clinical signs and levels of viral ribonucleic acid detection in the blood and oral fluid. The technique was capable of detecting classical swine fever virus nucleic acid as of day 7 post infection coinciding with the first detection in conventional oropharyngeal swab samples from some individual animals. Except for day 7 post infection in the “slower onset group”, the chances of classical swine fever virus nucleic acid detection in ropes were identical or higher as compared to the individual sampling.

**Conclusions:**

With the provided evidence, non-invasive oral fluid sampling at group level can be considered as additional cost-effective detection tool in classical swine fever prevention and control strategies. The proposed methodology is of particular use in production systems with reduced access to veterinary services such as backyard or scavenging pig production where it can be integrated in feeding or baiting practices.

**Electronic supplementary material:**

The online version of this article (doi:10.1186/s12917-016-0930-2) contains supplementary material, which is available to authorized users.

## Background

Early field detection of transboundary animal diseases (TADs) remains a key challenge within prevention and control efforts in the veterinary field. To overcome the shortage of timely information on the circulation of pathogens, oral fluid testing offers an opportunity to easily collect group-level disease data [1]. Over the past years, non-invasive sampling techniques based on the collection of oral fluid specimen have gained substantial importance in the field of swine herd health management. Aggregate level testing of oral fluid specimen has shown to be a valuable approach for increasing the efficiency and cost effectiveness of pathogen surveillance. For relevant, often endemic viral diseases affecting commercial swine production, such as porcine circovirus type 2, porcine reproductive and respiratory syndrome virus, and influenza A virus, protocols for sample collection and analysis are available [2]. However, the 2011 outbreak of foot and mouth disease (FMD) in Bulgaria, affecting mainly wildlife species, has highlighted the need for alternative surveillance methods at population level for TADs beyond the above mentioned [3]. This is in fact not only true for the wildlife sector, but also for farming structures where the access to the individual animal is limited, such as in certain backyard and scavenging production systems, where standard surveillance based on blood samples is challenging to implement.

A rope-in-a-bait sampling technique (referred to as pSWAB: “pathogen sampling of wild animals by baits”) for saliva collection of wild boar (*Sus scrofa*) has been developed and tested for its suitability to timely detect FMD virus shedding of experimentally infected animals [4]. For classical swine fever (CSF), due to the unexpected mild course of the infection during the experimental study on CSF in wild boar, detailed insights into the suitability of the sampling technique for early detection could not be obtained [5] and remained therefore speculative. The approach of non-invasive sampling for TADs was later picked up by Grau et al. [6] successfully detecting the shedding of viral genome of FMDV, ASFV and CSFV in oral fluids collected by the conventional chewing rope technique from infected animals using a multiplex RTqPCR. With CSF remaining one of the most relevant TADs for the pig sector at global level with dramatic socio-economic consequences for producers and other value chain stakeholders [7], the need for additional tools to improve field level disease information in the domestic pig population remains pertinent.

The aim of the present study was therefore to verify the assumed applicability of a recently developed, wild boar adapted, oral fluid sampling method for saliva collection and early CSF virus (CSFV) nucleic acid detection in domestic pigs.

## Methods

### Animal experiment

Eight pigs with a body weight of approximate 25 kg were purchased from commercial breeders, randomly separated in groups of four for management reasons and kept in a high containment isolation unit. Each animal was infected with a viral dose of 10^6^ tissue culture infectious dose 50% (TCID_50_) of CSFV wild type Alfort/Tübingen, administered intramuscularly. Starting from day −1, all animals were examined daily until day 15 post infection (pi) following the clinical scoring scheme by Mittelholzer et al. [8], with modifications as described by Tews et al. [9]. Body temperature was measured rectal and increased temperatures above 39,5 °C were regarded as fever. The scheme allowed a structured observation of the clinical course of the infection and to determine humane end points for the individual animal. Animals developing severe clinical signs, not exceeding defined end point criteria like fever over 42 °C, massive disorders of the CNS or bloody diarrhea were assigned to get slaughtered by legitimated staff of the Friedrich-Loeffler-Institute. To comply with the biorisk regulations of the research facility the slaughter extends to all pigs that survived until the end of the study.

### Sampling

For the oral fluid sample collection, eight to ten “pSWAB” sampling baits consisting of a raw cotton rope embedded in a cereal based bait matrix as described by Mouchantat et al. [5] were provided per pen on days −1, 2, 5, 7, 9, 12, and 15 pi. The “pSWABs” where distributed randomly within the pen by throwing them one-by-one over the pen barrier just avoiding the area the animals chose for defecation. Chewed on cotton ropes were collected from the pen flooring as found either at the same day or the next morning, incubated for one hour at room temperature in 3 ml of medium (Eagels’s minimum essential medium) of which aliquots of 1 ml were stored at −20 °C until further analysis. Blood samples were taken on days following the pSWAB distribution scheme using Monovette^®^ EDTA KE/9 ml (Sarstedt, Numbrecht, Germany) and stored at −70 °C until further analysis. Conventional oropharyngeal swab (Copan Rayon Regular Tip cat. no. 155C, Hain Lifescience GmbH, Nehren, Germany) samples were taken in parallel to the blood samples while animals were restrained. These were subsequently soaked in 1 ml of medium (Eagels’s minimum essential medium) and incubated for one hour at room temperature, aliquoted and stored at −20 °C until further analysis.

### Sample analysis

Extraction of RNA from all obtained samples was performed using the MagAttract Virus Mini M48 Kit for automated extraction (Qiagen GmbH, Hilden, Germany) according to the manufacturer’s recommendation. Specific CSF viral RNA detection was done through real time reverse transcription polymerase chain reaction (RTqPCR) according to the protocol of Hoffmann et al. [10]. Samples with C_q_ values below 40 were considered positive.

## Results

All animals developed clinical CSF but with differing degrees of severity (refer to Additional file 1 for details). One animal in group 1 had to be euthanized at day 8pi due to animal welfare reasons. All other animals remained in the experiment until day 15pi.

The pSWABs distributed in the pen were found to be attractive to the animals, they chewed on the ropes extensively and eventually just dropped them on the pen floor where they got picked up by another animal irrespectively of visible baiting matrix still present on the rope or not. It was observed, for each distribution, that the cotton ropes were chewed on by more than one animal of the group and sometimes animals chewed on more than one rope at the same time. The number ropes collected per sampling date for analysis varied between 4 and 9 per pen and represent the total number of visibly chewed ropes found. The ropes were collected manually from the pen flooring and did have slightly differing degrees of visible contamination depending on the location they got dropped by the last animal chewing on it. No ropes were found heavily contaminated with faeces and the subsequently obtained samples for analysis were visibly clear.

**Fig. 1:**
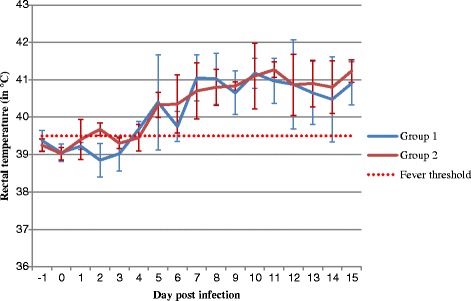
Mean rectal temperatures (°C) of the animals by group

The laboratory results of the oral fluid and blood analysis are summarized in Table 1. Within the applied sampling interval, CSFV nucleic acid was detectable in the blood starting from 2dpi in 2 out of 8 animals infected, and was evident in all animals on day 5pi. The detection in the blood lasted in all infected animals until the end of the experiment at day 15pi. The minor difference in the course of infection of the two equally treated groups is described by the faster onset of positive blood samples (starting from day 2pi) in combination with a first fever peak (see recorded rectal temperatures in Fig. 1) and overall lower C_q_ values over the course of the experimental period for animals in group 2.

**Table 1:** Viral RNA detection in domestic swine infected with viral doses of 10^6^ TCID_50_ CSFV Alfort/Tübingen

			Day post infection						
Group	Animal/sample	Sample Type	−1	2	5	7	9	12	15
1	1	EDTA blood	neg	neg	**33,76**	**32,24**	**27,48**	**27,6**	**27,22**
		OP swab	neg	neg	neg	neg	neg	neg	37,03
	2	EDTA blood	neg	neg	36,26	**30,03**	**29,61**	**28,3**	**29,57**
		OP swab	neg	neg	neg	39,01	**35,7**	neg	**35,95**
	3	EDTA blood	neg	neg	36,11	**30,14**	-	-	-
		OP swab	neg	neg	neg	neg	-	-	-
	4	EDTA blood	neg	neg	**34,35**	**32,01**	**28,26**	**26,5**	**25,77**
		OP swab	neg	neg	neg	neg	neg	36,7	**31,62**
	pSWAB	saliva	neg	neg	neg	neg	1(4)	9(9)	9(9)
		average Cq of positive					**34,46**	**33,1**	**28,98**
2	5	EDTA blood	neg	39,1	**27,27**	**24,18**	**21,49**	**20,1**	**19,78**
		OP swab	neg	neg	neg	neg	38	**33,2**	**31,36**
	6	EDTA blood	neg	**34,85**	**27,37**	**22,09**	**17,08**	**18,1**	**18,44**
		OP swab	neg	neg	neg	neg	**32,49**	**31,4**	**26,3**
	7	EDTA blood	neg	neg	**27,58**	**28,9**	**18,45**	**18,2**	**17,71**
		OP swab	neg	neg	neg	neg	**29,31**	**29,4**	**28,16**
	8	EDTA blood	neg	**34,4**	**27,11**	**21,3**	**17,74**	**16,9**	**16,41**
		OP swab	neg	neg	neg	**31,22**	**33,35**	**29,3**	**26,37**
	pSWAB	saliva	neg	neg	neg	4(8)	4(4)	4(4)	4(4)
		average Cq of positive				**33,82**	**30,23**	**28**	**28,74**

Cq values for EDTA blood, oropharyngeal (OP) swabs, pSWAB. Cq values ≥40 were considered negative, values between 36 and 40 doubtful and values <36 (in bold) positive. Counts for pSWABs are number of positive (total collected). (−): animal euthanized for animal welfare reasons

**Fig. 2:**
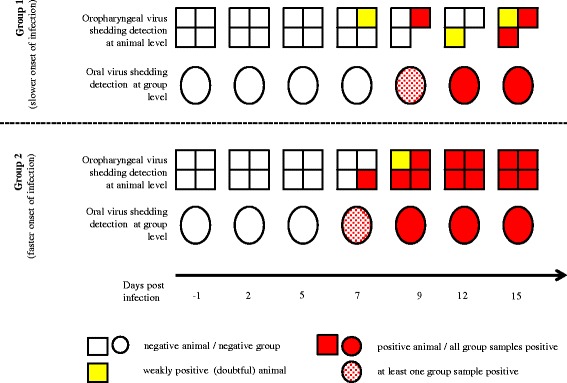
Comparison of CSFV nucleic acid detection in oral fluid collected at individual level (swab) or group level (pSWAB)

An overview on the level of CSFV nucleic acid detection in oral fluid collected at individual level compared to the group level sampling for both groups is given in Fig. 2. For group 1, on day 7pi one animal was tested doubtful in the oropharyngeal swab with all collected pSWABs remaining negative. Group 2 had one out of 4 animals tested positive on day 7pi with the oropharyngeal swab. At the same time 50% of the collected pSWABs (*n* = 4) tested positive. Throughout the remaining course of the experiment, CSFV specific RNA detection was always possible in all collected pSWABs, meanwhile the individual oropharyngeal testing of animals showed changing results in particular in group 1.

## Discussion

Building on the experiences made for oral fluid collection at population or herd level in wild boar [4], the present study was designed to determine the applicability of this rope-in-a-bait group based oral fluid sampling method for the detection of CSFV nucleic acid shedding during the course of a field virus infection of domestic pigs under controlled conditions. In a similar approach this has been tested in wild boar by Mouchantat et al. [5] but a sound evaluation of this non-invasive sampling methodology was not possible due to an unexpected mild course of the experimental infection. The results obtained later by Grau et al. [6] showed the feasibility of using the similar conventional rope sampling technique for CSFV genome detection but leaving out the “baiting component” essential for extensive production systems or wild boar sampling. In addition, focusing on the analytical sensitivity of the multiplex RTqPCR, this study did not deliver comparative values for blood samples and the different oral fluid samples over the course of infection.

The observed onset and duration of the detection of CSFV nucleic acid in blood samples in this study confirmed the existing knowledge on longer lasting viremic periods and CSFV specific RNA detection in blood of CSFV infected pigs [11]. Oropharyngeal shedding of CSFV nucleic acid was detectable starting from day 7pi and continued over the 15 day pi observation period, although in group 1 the shedding was not detectable continuously. These results are comparable to the findings made by Kaden et al. [12] for conventional oral swab testing in CSFV infected wild boar. Comparing these results with the detection of CSFV nucleic acid using the pSWAB, hence not targeting the individual animal but a group of animals housed together, the time window for virus genome detection is not differing.

The slightly differing course of the infection in the 2 groups, with group 1 showing a slower onset and lower levels of CSF viral RNA, brought up a difference in the initial detection of CSFV nucleic acid using the pSWAB. With only one very weakly positive animal (oral swab, Cq value close to negative cut off) in group 1 on day 7pi, the group level test was not sensitive enough. Apart from that circumstance, the group-level test showed similar or better sensitivity as the standard oropharyngeal swab sampling of individual animals (Fig. 2). The differing levels of excretion depending on the severity of the infection are in-line with findings from Weesendorp et al. [13]. The fact that the cotton ropes where collected from the pen flooring, hence an “open environment”, leaves room for potential cross contamination from other excreta of the animals in the group. This could to some extend lead to a false attribution of CSFV genome detection to saliva. Considering the results obtained by Weesendorp et al. [13] on virus detection in different excreta over the course of a CSF infection, this should not impact the onset of detection unless a rather substantial sample dilution takes place.

With the high variability of clinical signs that can be associated with CSF, pathogen detection remains an essential component of its diagnosis [14] confirming adequate field samples as an integral component of CSF surveillance that must not be compromised due to access problems. Despite the availability of good vaccines and robust diagnostic protocols, CSF control efforts often run into difficulties when dealing with pig populations less accessible to the implementing veterinary personnel [15]. As a consequence, alternative strategies for effective population or herd level monitoring of circulating viruses such as the pSWAB-methodology could assist in better targeting of field interventions in settings where sampling of individual animals is challenging, despite the reduced sensitivity and timeliness compared to blood sampling.

The acquired knowledge can on the one hand be useful for disease prevention strategies as well as surveillance tool within an immediate response to a CSF epidemic. On the other hand, it might be considered relevant for CSF surveillance in endemic settings. Countries with large proportions of pigs kept in disperse production systems such as free range, scavenging or certain types of backyard pig production tend to struggle in the collection of sound epidemiological field data on CSFV circulation. The collection of saliva at group level with a rope-in-a-bait method can easily be integrated in baiting or feeding regimes commonly practiced in extensive or scavenging pig production as well as in wild boar management. As the method applied does not rely on purity of oral fluids analyzed, moderate contamination with other body fluids or faeces are not likely to influence the results negatively.

However, case studies in the field will be needed to identify realistic best practices in the timely backflow of recovered cotton ropes to the laboratories capable of conduction CSFV nucleic acid detection in particular looking into the suitability of oral fluid samples as sampling material under these distinct field conditions. In particular, the overall environmental impact through temperature and humidity on virus genome stability will need to be evaluated when applying this method. This needs fine-tuning of the approach to specific local conditions in CSF endemic countries before considering it for larger scale implementation.

## Conclusions

The importance to expand the methodologies of non-invasive sampling for the surveillance of some of the major TADs that can affect suids became once more evident in the 2011 FMD outbreak in Bulgaria [3]. For host populations with limited access to the individual animal sample acquisition will need to focus on group-level testing. It can be concluded that under experimental conditions, the described “rope-in-a-bait” sampling technique has confirmed its suitability in principle to detect circulating CSFV and can potentially lead to easier and cheaper field sample collection leading to overall more cost-effective surveillance in these specific target population.

## Additional file

Additional file 1: Body temperature and clinical score of the individual animals through the course of the experiment. (XLSX 24 kb)

## Abbreviations

CSF: Classical swine fever
CSFV: Classical swine fever virus
FMD: Foot and mouth disease
pi: Post infection
pSWAB: Pathogen sampling of wild animals by baits
RTqPCR: Real time reverse transcription polymerase chain reaction
TADs: Transboundary animal diseases
TCID_50_: Tissue culture infectious dose 50%
